# Strategic Carbon Source Selection Enhances Biomass and Paramylon Yields in Mixotrophic *Euglena gracilis* Cultivation

**DOI:** 10.3390/microorganisms13102339

**Published:** 2025-10-11

**Authors:** Xue Xiao, Rui He, Xinyue Guo, Xinxin Zhao, Zhengfei Yang, Yongqi Yin, Minato Wakisaka, Jiangyu Zhu

**Affiliations:** 1 School of Food Science and Engineering, Yangzhou University, Yangzhou 225127, China; mz120232138@stu.yzu.edu.cn (X.X.); 17313339720@163.com (R.H.); mz120242221@stu.yzu.edu.cn (X.G.); xxzhao@yzu.edu.cn (X.Z.); yzf@yzu.edu.cn (Z.Y.); yqyin@yzu.edu.cn (Y.Y.); 2 Food Study Centre, Fukuoka Women’s University, 1-1-1 Kasumigaoka, Fukuoka 813-8529, Japan

**Keywords:** *Euglena gracilis*, organic carbon sources, cell morphology, β-1,3-glucan, photosynthetic pigment synthesis

## Abstract

*Euglena gracilis*’s mixotrophic metabolism offers biotechnological potential. This study investigated how glucose, sodium acetate, ethanol, and propanetriol regulate its growth, photosynthesis, and paramylon production. All carbon sources boosted paramylon yield versus photoautotrophic controls. Ethanol and glucose were both highly effective, supporting the highest biomass accumulation (5.71 and 4.42-fold increases, respectively) and paramylon content without a significant difference between them. Ethanol supplementation enhanced chlorophyll b via coupled TCA cycle/glyoxylate shunt activity, while glucose showed the strongest tendency for high paramylon and the highest carotenoid content (13.36-fold higher). Sodium acetate triggered alkaline stress (pH 8.5), suppressing pigments and inducing spherical cells. Propanetriol reduced biomass but enhanced PSII efficiency (Fv/Fm). These results demonstrate carbon source-driven metabolic partitioning: ethanol and glucose both excel in promoting growth and storage, while additionally directing carbon toward chlorophyll b or carotenoids, respectively. These findings enable targeted bioprocess optimization: selection between ethanol or glucose can be based on the value of co-products, advancing *E. gracilis* as a sustainable cell factory.

## 1. Introduction

Organic carbon sources play a critical role in microalgal cultivation [1,2]. They provide essential energy for cellular growth and metabolism and serve as fundamental building blocks for synthesizing vital biomolecules. The efficiency of organic carbon utilization directly determines the microalgae’s biomass accumulation and high-value product synthesis, impacting the economic viability and practical applications of the cultivation process [3,4]. *Euglena gracilis* is a distinctive microalga exhibiting remarkable environmental adaptability due to its capability for autotrophic, heterotrophic, and mixotrophic growth [5,6]. Under autotrophic conditions, *E. gracilis* uses light energy via chloroplasts for photosynthesis, con-verting carbon dioxide and water into organic matter while releasing oxygen [4]. This process supplies energy for growth and facilitates the accumulation of storage compounds like β-1,3-glucan. In heterotrophic mode, when light is limited or organic carbon is abundant, *E. gracilis* absorbs external organic compounds (e.g., sugars, organic acids, amino acids) through membrane transporters, metabolizing them for energy and carbon skeletons [7]. Mixotrophic growth combines both strategies, enabling simultaneous CO_2_ fixation and organic carbon uptake [8]. This synergy significantly enhances the alga’s growth and metabolic rates in challenging environments [5].

Importantly, the type of organic carbon source profoundly influences the metabolic partitioning and end-product spectrum in *E. gracilis* [9]. This differential regulation stems from their distinct pathways of assimilation and integration into core metabolism, as conceptually summarized in the graphical abstract. Briefly: glucose, as a hexose, is readily assimilated and rapidly enters glycolysis, providing rapid energy (ATP), reducing power (NADPH), and carbon skeletons (e.g., glucose-6-phosphate) that can be channeled toward both biomass proliferation and the synthesis of storage carbohydrates like paramylon. Sodium acetate is activated to acetyl-CoA, primarily fueling the tricarboxylic acid (TCA) cycle for energy generation and precursor supply; its anaplerotic role can enhance precursor availability for paramylon synthesis [10,11]. Ethanol passively diffuses into cells and is oxidized to acetaldehyde and then acetyl-CoA, similarly entering the TCA cycle; its metabolism is often coupled with the glyoxylate shunt, making it particularly efficient for biomass and chlorophyll production under mixotrophy. Propanetriol, a triose, requires ATP-dependent phosphorylation and oxidation to enter glycolysis as dihydroxyacetone phosphate (DHAP), resulting in a slower metabolic flux that can favor the accumulation of neutral lipids [12]. Therefore, strategically selecting and optimizing the carbon source concentration based on the specific cultivation objective—whether maximizing biomass, β-1,3-glucan, or lipids—is crucial for refining *E. gracilis* cultivation and boosting target product yields.

Although research on organic carbon utilization in microalgae, including *E. gracilis*, has progressed—identifying preferences like glucose for enhancing cell density and oil content in *Scenedesmus obliquus* and *Chlorella* sp. [2], propanetriol for *Phaeodactylum tricornutum* [13], and biogas-derived carbon for boosting Spirulina biomass [13]—these findings support theoretical frameworks and industrial fermentation optimization. However, a systematic and in-depth understanding of how specific carbon sources affect the physiological and biochemical characteristics of *E. gracilis* remains limited [14]. This knowledge gap significantly hinders efforts to improve its industrial-scale cultivation efficiency.

Therefore, this study aims to systematically investigate the effects of key organic carbon sources (glucose, sodium acetate, propanetriol, ethanol) on the core physiological and biochemical traits of *E. gracilis*. We will analyze growth dynamics, photosynthetic and respiratory activity, and β-1,3-glucan synthesis under different carbon source conditions. Through integrated analysis, we seek to elucidate the molecular mechanisms by which organic carbon sources regulate the synthesis and accumulation of high-value products, particularly β-1,3-glucan, in *E. gracilis*. This research is expected to advance the theoretical understanding of mixotrophic metabolism in this alga, offering new insights for microalgal metabolic research. Practically, it will provide a scientific foundation for optimizing high-yield cultivation, reducing production costs, and enabling targeted biosynthesis of valuable compounds. These outcomes will facilitate the large-scale application of *E. gracilis* in food, feed, bioenergy, and pharmaceutical industries.

## 2. Materials and Methods

### 2.1. Experimental Design

#### 2.1.1. Algae Strains and Culture Medium

*Euglena gracilis* FACHB-850 was purchased from Freshwater Algae Culture Collection at the Institute of Hydrobiol-ogy, Chinese Academy of Sciences (Wuhan, China). The strain was cultured in CM medium [15].

#### 2.1.2. Carbon Source Addition and Sterilization

Based on the total organic carbon (TOC) concentration of 40 mmol/L, according to the molecular formula of carbon source, the addition amount was calculated as follows: glucose (C_6_H_12_O_6_) (CAS 50-99-7, Catalog #10010518, ≥99.5%) 6.67 mmol/L, sodium acetate (C_2_H_3_O_2_Na) (CAS 127-09-3, Catalog #10018818, ≥99.0%) 20 mmol/L, ethanol (C_2_H_5_OH) (CAS 64-17-5, Catalog #10009218, ≥99.7%) 20 mmol/L, propanetriol (C_3_H_8_O_3_) (CAS 56-81-5, Catalog #10010618, ≥99.0%) 13.33 mmol/L. Glucose, sodium acetate and propanetriol were autoclaved (115 °C, 15 min), and ethanol was filtered through a 0.22 μm filter membrane.

#### 2.1.3. Culture Conditions

The algae were pre-cultured to the logarithmic growth phase (density ≥ 1 × 10^6^ cells/mL, determined using a Neubauer-improved hemocytometer (0.100 mm depth, 0.0025 mm^2^ area; Paul Marienfeld GmbH & Co. KG, Lauda-Königshofen, Germany)), and inoculated into a 300 mL conical flask containing 180 mL medium (breathable silicone plug) at an initial density of 1 × 10^4^ cells/mL, corresponding to an initial dry weight of 4.0 ± 0.5 mg/L, according to 20 mL inoculation amount. Culture conditions: temperature (25 ± 2) °C, light-dark cycle 12 h:12 h (white LED, 5000 lx, vertical unilateral irradiation), hand-shaking twice a day (09:00–10:00 and 16:00–17:00) to avoid cells adhesion [16]. The control group was cultured under identical conditions but without the addition of any organic carbon source, representing a photoautotrophic baseline. Based on extensive preliminary experimentation, the cultivation period was established at 10 days. This duration was deemed adequate for the cultures to reach the stationary phase and attain near-maximal biomass and paramylon yield under the specified conditions, thus enabling a valid comparison of endpoint productivity across all carbon source treatments.

### 2.2. Evaluation of Cell Growth of E. gracilis

The algae liquid of each group was collected on the 10th day of culture, by which time all cultures had reached the stationary growth phase as determined by preliminary experiments showing that optical density at 680 nm (OD_680_) plateaued beyond this point. The empty beaker was pre-treated by a blast drying oven (105 °C) to constant weight, and then placed in a dryer (silica gel) for cooling for 30 min and weighed (recorded as W_1_) [17]. The 8 mL algae solution was taken in a 10 mL centrifuge tube, symmetrically balanced with deionized water, centrifuged at 8000× *g* for 10 min, and the supernatant was removed. The precipitate was resuspended with deionized water and washed twice. The algae residue was quantitatively transferred to a pre-weighed, heat-resistant glass beaker (W_1_) with 1 mL deionized water. The beaker was chosen for its chemical inertness, stability at 105 °C, and sufficiently large surface-to-volume ratio to facilitate efficient moisture evaporation. The sample was dried to constant weight at 105 °C (typically requiring 24–48 h with periodic checking) and weighed after cooling in a desiccator (W_2_). Achieving constant weight (i.e., successive weighings differing by less than 0.1 mg) ensured complete water removal and validated the use of this container. Biomass dry weight was calculated according to the formula:

Dry weight (mg/L) = (W_2_ − W_1_)/0.008 (1)

### 2.3. Determination of pH of E. gracilis Medium

To prevent ongoing metabolic activity (e.g., respiration, photosynthesis) from altering the pH during the measurement, it was critical to separate the cells from the culture medium prior to measurement. Every 2 days, 3 mL of algae solution was centrifuged at 10,000× *g* for 15 min. The pH of the cell-free supernatant was then immediately measured. To confirm that the centrifugation process itself did not significantly alter the pH, a control experiment was performed where the pH of unfiltered/uncentrifuged culture media was compared to that of centrifuged media; no significant difference was found (*p* > 0.05).

For the measurement, the pH meter (PHS-2F, Shanghai Yeidian Technology Co., Ltd., Shanghai, China) was calibrated with pH 4.00 and 6.86 standard buffer solutions. The electrode was inserted 1 cm below the liquid level of the centrifugal supernatant. After the reading stabilized within 30 s, it was recorded. This process was repeated three times, and the average value was used as the pH value of the sample.

### 2.4. Evaluation of Cell Morphology of E. gracilis

Cell morphology is a key indicator to evaluate the regulatory effect of exogenous carbon source metabolism. Under different environmental conditions, *E. gracilis* may exhibit spherical, spindle-shaped, slender and other diverse forms [18]. On the 10th day of culture (stable period), the living algae droplets were added to the slides, and the coverslips were im-mediately observed using an optical microscope. After scale calibration and contrast optimization, the images were im-ported into ImageJ software (version 1.53t; National Institutes of Health, USA), and the cell contour was manually selected to measure the aspect ratio. Three biological replicates were measured in each group.

### 2.5. Determination of Photosynthetic Pigments of E. gracilis

According to the method of Lichtenthaler [19], 10 mL algae solution was taken on the 10th day of culture, filtered by 0.45 μm nylon filter membrane, and transferred to the mortar after filter cake. Appropriate amount of quartz sand and 80% (*v*/*v*) acetone were added and ground for 5 min. The homogenate was filtered through a filter paper (or a sintered glass funnel), and the residue was thoroughly rinsed with 80% (*v*/*v*) acetone until it became colorless. The combined filtrates were then diluted to a final volume of 10 mL with 80% acetone in the dark. A_470_, A_646_ and A_663_ were determined by ultraviolet-visible spectrophotometer with 3 mL extraction solution and 80% acetone as blank. Calculate the pigment yield according to the following formula:

Chlorophyll a production = 12.21 × A_663_ − 2.81 × A_646_ (2)

Chlorophyll b production = 20.13 × A_646_ − 5.03 × A_663_ (3)

Carotenoid production = (1000 × A_470_ − 3.27 × Chla − 104 × Chlb)/229 (4)

The pigment production, quantified in mg/L using Equations (2)–(4), underwent subsequent conversion to a percentage of dry weight (% DW). The dry weight of the biomass was ascertained as detailed in Section 2.2. The pigment content was computed via the following formula: Pigment content (% DW) = [Pigment production (mg/L)]/[Biomass dry weight (mg/L)] × 100%. This methodology mirrors the calculation employed for paramylon content, as presented in Section 2.7.

### 2.6. Determination of Chlorophyll Fluorescence Parameters of E. gracilis

Chlorophyll fluorescence kinetics effectively capture photosynthetic responses to carbon source stress. Based on observed pigment variations, we measured: PSII maximum photochemical efficiency (F_v_/F_m_); potential activity (F_v_/F_o_); energy allocation parameters (per reaction center): light absorption (ABS/RC), thermal dissipation (DIo/RC), trapping efficiency (TRo/RC), and electron transport efficiency (ETo/RC). These metrics clarify carbon source regulation of photosynthetic energy conversion.

On day 10 of cultivation (stationary phase), 3 mL of algal culture was collected from each group and subjected to dark adaptation for 30 min under the condition of avoiding light, and the determination was carried out by hand-held pulse modulated fluorescence spectrometer (FluorPen FP110, Photon Systems Instruments, Drásov, Czech Republic). Three biological replicates were set in each group, and all parameters were collected synchronously after a single dark adaptation [20]. Although measurements were conducted at the stationary phase, they robustly reflect the sustained physiological status and long-term adaptive responses of the photosynthetic apparatus to the different carbon source regimes.

### 2.7. Quantification of β-1,3-Glucan in E. gracilis

Modified from Barsanti et al. [21], on day 10 of cultivation, 25 mL algal culture was centrifuged at 8000× *g* for 15 min. Cell pellets were collected via three sequential centrifugation cycles. Pellets were frozen at −20 °C for 12 h, then suspended in 5 mL extraction buffer (50 mM Tris-HCl, 1% SDS). Samples were sonicated (40 kHz, 250 W, 15 min) followed by 1 h incubation at 37 °C. The mixture was centrifuged (8000× *g*, 30 min). Supernatants were discarded until they were clear, and pellets were washed thrice with 70 °C deionized water. Washed samples were transferred to pre-weighed beakers (W_1_) and dried to constant weight at 80 °C under forced-air drying. After 30 min desiccator cooling, final weight (W_2_) was recorded. Calculation:

β-1,3-glucan production (mg/L) = (W_2_ − W_1_)/0.025 (5)

β-1,3-glucan content (% DW) = [β-1,3-glucan production]/[Biomass dry weight] × 100(6)

### 2.8. Statistical Analysis

Statistical analyses were performed using SPSS 26.0 (IBM Corp., Armonk, NY, USA). The Shapiro–Wilk test was used to assess the assumption of normality, and Levene’s test was used to assess the homogeneity of variances. One-way analysis of variance (ANOVA) was used to compare differences among groups for data that met both assumptions (normality and homoscedasticity, both with *p* > 0.05). The non-parametric Kruskal–Wallis test was used for data that violated either or both of these assumptions. Post hoc multiple comparisons were performed using Tukey’s Honest Significant Difference (HSD) method for parametric data for groups that showed significant differences (*p* < 0.05). Mean ± standard deviation (SD) is used to present graphical data. All visualizations were generated using Origin 2021 (version 2021, OriginLab Corporation, Northampton, MA, USA).

## 3. Results and Discussion

### 3.1. Effects of Organic Carbon Sources on the Growth, Dry Weight and pH of E. gracilis

**Figure 1:**
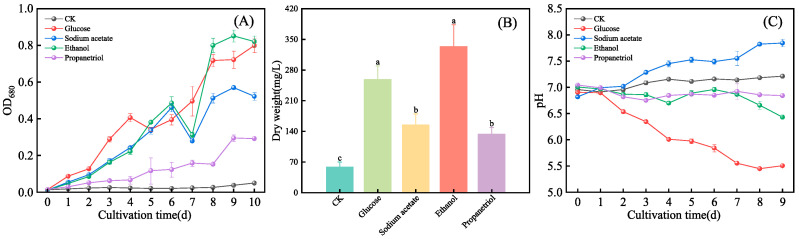
The biomass and pH of *E. gracilis* under different organic carbon. (**A**) OD_680_; (**B**) Dry weight measured at the end of the 10-day cultivation period; (**C**) pH. Data are presented as mean ± SD (*n* = 3). Different lowercase letters indicate statistically significant differences among treatments (*p* < 0.05, one-way ANOVA with Tukey’s HSD test). In Figure 1, consistent but distinct color schemes are employed for different data types to optimize clarity: a sequential color palette for the time-series data (line graphs, panels (**A**,**C**)) and a categorical color palette for the comparative group data (bar graph, panel (**B**)). This approach follows data visualization best practices to most accurately represent the respective data structures.

As shown in Figure 1A, cell growth rates of *E. gracilis* under four organic carbon sources and the control group showed no significant differences during days 0–2, indicating an adaptation phase to new environmental conditions. After the adaptation phase, distinct growth-promoting effects emerged. Under identical autotrophic conditions supplemented with organic carbon sources, the mean biomass yield was highest in the ethanol treatment (16.54-fold increase vs. control) and high in the glucose treatment (16.09-fold increase); however, this difference was not statistically significant (*p* > 0.05). Yields in the sodium acetate and propanetriol treatments were significantly lower (10.52-fold and 5.89-fold increases, respectively). These results demonstrate that carbon source utilization efficiency directly governs microbial growth performance [22].

As a monosaccharide, glucose facilitates rapid energy metabolism and cell proliferation. Sodium acetate requires transmembrane transport and metabolic conversion, causing delayed utilization. Concomitant pH elevation further inhibits transporter activity and acetate uptake [23]. Ethanol passively diffuses into cells due to its small molecular size, with enzymatic conversion boosting utilization efficiency post-adaptation [24]. Propanetriol necessitates complex phosphorylation (via propanetriol kinase) to glyceraldehyde-3-phosphate, resulting in low metabolic flux and limited growth stimulation [25]. Consequently, glucose and ethanol emerged as the most effective exogenous organic carbon sources.

Dry weight, a key biomass indicator for *E. gracilis*, exhibited significant variation across treatments (Figure 1B). Com-pared to the control group (58.33 mg/L), all organic carbon sources enhanced biomass accumulation (*p* < 0.05). Ethanol and glucose showed the strongest effects, with dry weights reaching 333.33 mg/L (5.71-fold increase) and 258.33 mg/L (4.42-fold increase), respectively. Sodium acetate and propanetriol treatments yielded 154.17 mg/L (2.64-fold) and 133.33 mg/L (2.28-fold). Together with the dry weight data for ethanol and glucose, the statistical analysis revealed two distinct effectiveness groups: (1) ethanol and glucose, which were statistically equal and yielded the highest biomass; and (2) sodium acetate and propanetriol, which were also statistically comparable but significantly less effective than the first group. These results validate that mixotrophic cultivation outperforms autotrophic growth in biomass production, providing a basis for process optimization [26].

It is important to note that growth monitoring via optical density (OD_680_) may be influenced by concurrent alterations in cellular morphology (e.g., cell size and shape impacting light scattering) and pigment composition (e.g., modifying absorbance at specific wavelengths), which are established microalgal responses to varying carbon sources. Nevertheless, the strong correlation between OD_680_ temporal trends and final dry cell weight measurements across all treatments (Figure 1A vs. Figure 1B) suggests that OD_680_ provided a robust and reliable metric for assessing relative growth dynamics and biomass accumulation under our experimental conditions. The specific morphological and pigmentary changes induced by each carbon source are quantified and discussed in subsequent sections.

pH fluctuations exhibited significant variability across treatments, correlating with distinct metabolic processes (Figure 1C). The most pronounced acidification was observed in the glucose treatment, where the pH decreased rapidly from 7.0 to 6.54 within the first 2 days, and continued to decline to 5.45 by day 9. This trend is likely due to accelerated cell proliferation, leading to increased CO_2_ production and NH_4_^+^ ion consumption [27]. Conversely, cultures supplemented with sodium acetate became alkaline, with the pH rising to 7.29 by day 2 and remaining elevated (between 7.45 and 7.85) from day 4 until the end of the cultivation. This sustained alkalinity is attributed to its weak-acid/strong-salt characteristics and was likely amplified by reduced algal growth under non-optimal pH conditions. The ethanol treatment maintained a near-neutral pH (between 6.86 and 7.29) for the first 4 days before gradually acidifying to 6.43 by day 9, potentially due to the accumulation of respiratory CO_2_ post-adaptation [28]. Lastly, the propanetriol treatment displayed the most stable pH, fluctuating within a narrow range of 6.82 to 7.04 throughout the entire 10-day experiment, which may be a result of neutral byproduct generation during its metabolism [26].

Although the growth trend for glucose on day 10 suggested potential for further increase (Figure 1A), the 10-day endpoint was selected as the standard cultivation period for all treatments to ensure a consistent and comparable measure of productivity. This decision was based on the attainment of stationary phase in the majority of cultures and the primary research objective of comparing the efficiency of carbon source utilization under a standardized timeframe, rather than optimizing the harvest time for each individual carbon source.

### 3.2. Effects of Different Organic Carbon Sources on the Cell Morphology of E. gracilis

Cell morphology analysis (Table 1) revealed significant carbon source-dependent effects. The control group exhibited an aspect ratio of 2.513 ± 0.340. Glucose supplementation increased this ratio to 3.697 ± 0.729 (*p* < 0.01), though with high intragroup variability. In contrast, sodium acetate reduced the ratio to 1.541 ± 0.341 (*p* < 0.01), producing uniformly shortened cells. Ethanol induced the highest elongation (4.224 ± 1.104, *p* < 0.01), but extreme data dispersion indicated heterogeneous cellular responses. Propanetriol moderately increased the ratio to 3.593 ± 0.975 (*p* < 0.05), suggesting weaker elongation effects than glucose, likely linked to metabolic fluctuations.

**Figure 2:**
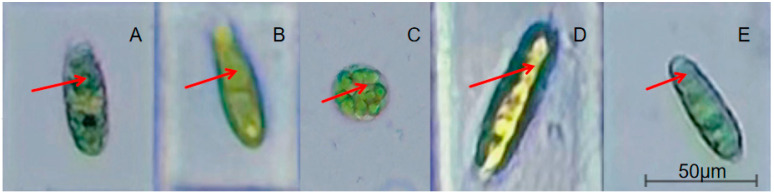
Effects of different carbon sources on cell morphology. Bright-field micrographs of *E. gracilis* cells from (**A**) control, (**B**) glucose, (**C**) sodium acetate, (**D**) ethanol, and (**E**) propanetriol treatments. The opaque, highly refractive intracellular granules indicated by arrows are characteristic of paramylon (β-1,3-glucan) granules. This identification is based on their distinctive morphology and high refractivity under bright-field microscopy, which is a well-established method for visualizing this storage polysaccharide in *E. gracilis* cells [21].

**Figure 3:**
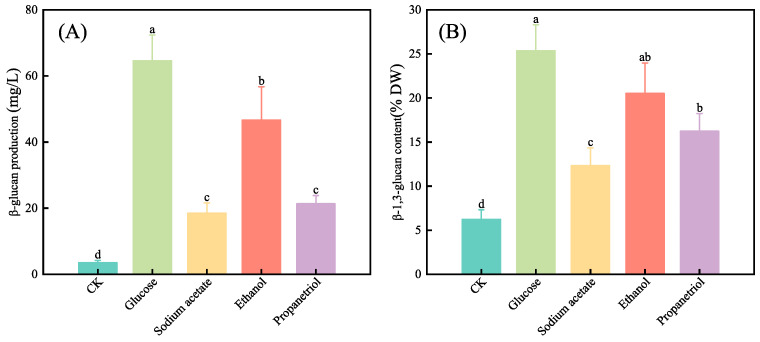
β-1,3-glucan production and content of *E. gracilis* under different organic carbon after 10 days of cultivation. (**A**) β-1,3-glucan production (mg/L); (**B**) β-1,3-glucan content (% DW). Different lowercase letters indicate statistically significant differences among treatments (*p* < 0.05, one-way ANOVA with Tukey’s HSD test).

Cell morphology in *E. gracilis* reflects physiological status and metabolic activity (Figure 2). Under varying carbon sources, length-to-width ratios correlated with growth traits, ranked in descending order: ethanol > glucose > propanetriol > control (CK) > sodium acetate. Ethanol group, elongated cells (highest aspect ratio) enhanced nutrient uptake and carbon metabolism via increased surface-area-to-volume ratios, complementing ethanol’s efficient utilization. Sodium acetate group, alkaline conditions from weak acid salts of strong bases induced spherical cell contraction (lowest ratio), minimizing mem-brane damage by reducing surface exposure. Elongation represents an adaptive strategy for high metabolic efficiency, while spherical morphology mitigates alkaline stress. These findings establish cell shape as a biomarker for assessing algal physiology [29]. The observed morphological changes were accompanied by variations in paramylon accumulation (as quantified in Figure 3), with the spherical cells induced by sodium acetate (Figure 2C) containing numerous large paramylon granules, consistent with their high intracellular paramylon content.

### 3.3. Effects of Different Organic Carbon Sources on Photosynthetic Pigments of E. gracilis

**Figure 4:**
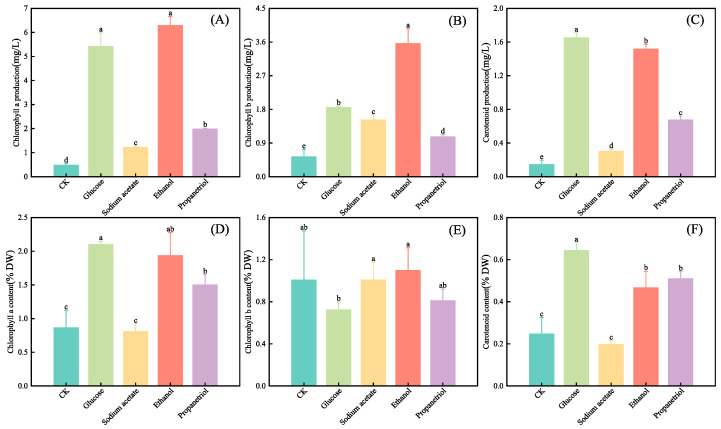
Photosynthetic pigments of *E. gracilis* under different organic carbon after 10 days of cultivation. (**A**) Chlorophyll a production (mg/L); (**B**) Chlorophyll b production (mg/L); (**C**) Carotenoid production (mg/L); (**D**) Chlorophyll a content (% DW); (**E**) Chlorophyll b content (% DW); (**F**) Carotenoid content (% DW). (**A**–**C**) show the photosynthetic pigment production (mg/L) determined by spectrophotometry; (**D**–**F**) show the pigment content as a percentage of cell dry weight (% DW). For detailed methods, see Section 2.5. Data are presented as mean ± SD (*n* = 3). Different lowercase letters indicate statistically significant differences among treatments (*p* < 0.05, one-way ANOVA with Tukey’s HSD test).

Photosynthetic pigment production (Figure 4) was quantified across treatments, including chlorophyll a, chlorophyll b, and carotenoid levels. Glucose and ethanol treatments yielded the highest pigment concentrations (chlorophyll a, b, and carotenoids) among all groups, indicating superior carbon utilization efficiency. This enhanced metabolic activity likely stimulated algal growth and pigment biosynthesis [30]. All treatment groups exhibited significant pigment increases compared to the control (*p* < 0.05). The effects of organic carbon sources on photosynthetic pigment production were complex and pigment-specific (Figure 4A–C). Ethanol treatment yielded the highest chlorophyll b content, which was significantly greater than all other groups (*p* < 0.05). In contrast, for chlorophyll a, both ethanol and glucose were equally effective, yielding concentrations that were significantly higher (*p* < 0.05) than those in the acetate, propanetriol, and control groups but not significantly different from each other (*p* > 0.05). Surprisingly, glucose supplementation resulted in the highest carotenoid content, which was significantly higher (*p* < 0.05) than that achieved with ethanol. This indicates that while both ethanol and glucose are highly effective carbon sources, their impacts on different branches of the photosynthetic pigment synthesis pathway are distinct.

Chlorophyll a and carotenoid levels varied significantly across treatments compared to the control (*p* < 0.05), whereas chlorophyll b remained stable. These differences stemmed from carbon source metabolism and stress-response mechanisms [31]. Glucose, enhanced chlorophyll a synthesis via glycolysis and the TCA cycle (providing energy and precursors) and activated carotenoid pathways, yielding the highest pigment accumulation [32]. Ethanol induced mild stress, promoting chlorophyll a synthesis and antioxidant-driven carotenoid accumulation [33,34], though partial metabolic inhibition counterbalanced these effects. Propanetriol, stable metabolism supplied reducing power for moderate carotenoid production [12]. Its gradual carbon release resulted in an intermediate chlorophyll a level (between ethanol and CK groups). Sodium acetate, alkaline conditions suppressed pigment-synthesizing enzymes and diverted resources to basal metabolism, minimizing both chlorophyll a and carotenoid content [35,36].

The significant alterations in pigment composition documented, specifically the notable elevation in the carotenoid-to-chlorophyll ratio in ethanol-cultivated cells, validate the potential technical concern associated with OD_680_ measurements. The absorption spectrum of carotenoids (400–500 nm) exhibits minimal overlap with the 680 nm wavelength employed for growth monitoring. Consequently, the strong correlation between OD_680_ and dry weight (Figure 1) implies that biomass increase was the primary determinant influencing OD_680_, surpassing spectral interference from pigment variations. This observation supports the reliability of our initial growth curves.

### 3.4. Effects of Different Organic Carbon Sources on Chlorophyll Fluorescence of E. gracilis

**Figure 5:**
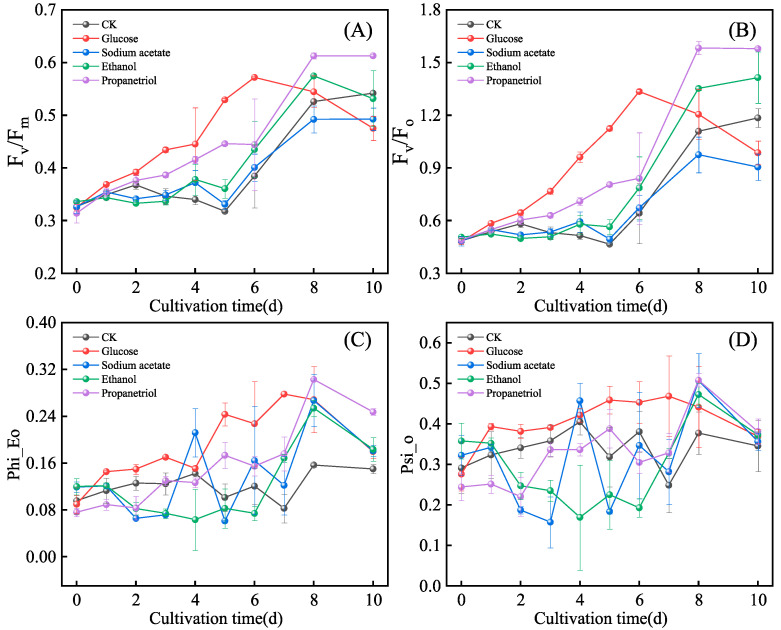
Basic efficiency parameters of photosystem II (PSII) of *E. gracilis* under different organic carbon. (**A**) F_v_/F_m_; (**B**) F_v_/F_o_; (**C**) Phi_Eo; (**D**) Psi_o.

PSII photochemical efficiency (F_v_/F_m_, reflecting light-energy conversion) and quantum yield (F_v_/F_o_, indicating potential photochemical activity) are key indicators of PSII functionality and algal physiological status. Higher plants typically exhibit F_v_/F_m_ values > 0.7, whereas microalgae show lower values due to evolutionary adaptations: (1) PSII repair mechanisms are less efficient in microalgae, leading to cumulative photodamage; (2) nutritional flexibility (hetero-trophic/mixotrophic capabilities) reduces reliance on photosynthesis, unlike obligatory phototrophic higher plants. These adaptations highlight trade-offs between metabolic versatility and photosynthetic optimization. This divergence under-scores evolutionary strategies across taxa and provides insights into microalgal environmental adaptability [37]. As shown in Figure 5A,B, the propanetriol group showed the highest F_v_/F_m_ and F_v_/F_o_ values, indicating superior PSII light-energy conversion and activity. In contrast, the sodium acetate group showed the lowest values (Figure 5A,B), suggesting a suppression of PSII functionality. This statistical conclusion is further supported by the distinct correlation between intermediate metabolite concentrations (glucose and ethanol) and exhibited a distinct correlation with the carbon source, indicating that organic carbon availability may exert a substantial influence on the photosynthetic apparatus. This influence may occur through direct metabolic regulation and indirect effects, such as alterations in culture density and light availability.

To rigorously quantify the differences in photosynthetic performance achieved by the end of the cultivation period, a statistical comparison of the F_v_/F_m_ and F_v_/F_o_ values at Day 10 was performed. This analysis confirmed significant disparities among the treatments (*p* < 0.05). The propanetriol group yielded the highest values (F_v_/F_m_: 0.613 ± 0.002; F_v_/F_o_: 1.579 ± 0.007), which were statistically greater than those of all other groups (*p* < 0.05). Conversely, the sodium acetate treatment resulted in the lowest values (F_v_/F_m_: 0.493 ± 0.021; F_v_/F_o_: 0.905 ± 0.075), which were significantly lower than those of the control, glucose, and ethanol groups (*p* < 0.05). The glucose and ethanol groups exhibited intermediate and statistically comparable values to each other (*p* > 0.05) but were significantly higher than the acetate group. These endpoint data robustly support the trends observed in Figure 5A,B, confirming that carbon source type definitively alters the maximum photochemical capacity of PSII in *E. gracilis*.

Phi_Eo quantifies the initial quantum yield of light-energy capture by PSII reaction centers, reflecting their intrinsic photochemical efficiency. Psi_o represents the probability of electron transfer from PSII to PSI after light-energy absorption. As shown in Figure 4D, no significant differences in Psi_o were observed across treatments (*p* > 0.05). Phi_Eo values in treatment groups exceeded those in the control, indicating that organic carbon sources enhance photosynthetic performance by activating mixotrophic metabolism. In Mechanism 1, organic carbon alleviates carbon limitation, supplying pre-cursors for chlorophyll and light-harvesting complex biosynthesis, thereby directly improving light-energy capture. In Mechanism 2, heterotrophic metabolism redistributes energy demands, reduces photoinhibition, and enhances antioxidant defenses, preserving PSII integrity. Concurrently, accelerated cell proliferation extends peak photosynthetic activity.

**Figure 6:**
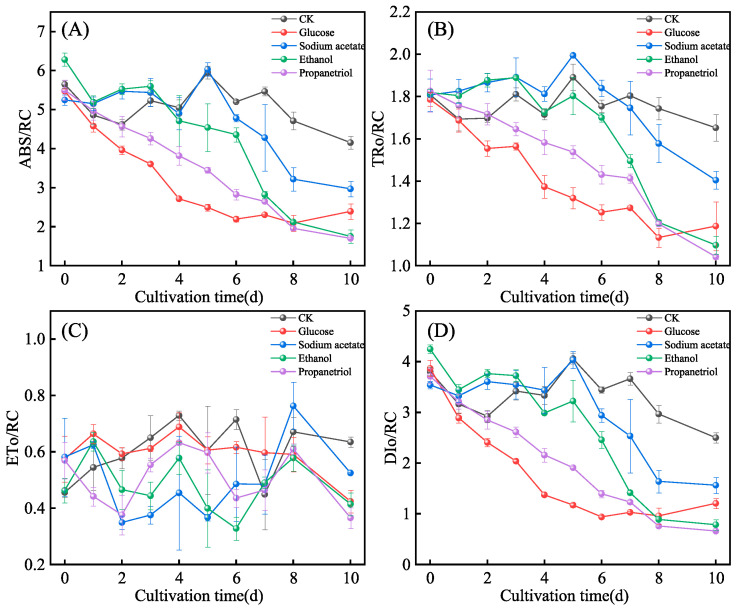
Energy distribution parameters of reaction of *E. gracilis* under different organic carbon. (**A**) ABS/RC; (**B**) TRo/RC; (**C**) ETo/RC; (**D**) DIo/RC.

Chlorophyll fluorescence parameters (ABS/RC: light absorption; DIo/RC: thermal dissipation; TRo/RC: electron transport; ETo/RC: transport yield) reflect the efficiency of light-energy absorption, allocation, and conversion in photo-synthetic machinery. ABS/RC (light absorption per reaction center), DIo/RC (thermal dissipation), and TRo/RC (electron transport) followed a descending order: CK > Sodium acetate > Glucose > Ethanol > Propanetriol (Figure 6A–D), indicating reduced light-energy capture, dissipation, and electron transport as carbon sources shifted from phototrophy (Control) toward heterotrophic dominance. ETo/RC (electron transport yield) exhibited a fluctuating pattern with the same hierarchy, high-lighting carbon source-dependent variations in electron transport efficiency. Propanetriol group, lowest ABS/RC and DIo/RC values, likely due to rapid cellular uptake of propanetriol (a small-molecule carbon source) supporting Calvin cycle activity. This stabilizes thylakoid membranes and enhances antioxidant defenses, mitigating photodamage and improving light-use efficiency. Sodium acetate group, high ABS/RC but low TRo/RC suggests acetate-induced disruption of proton gradients across membranes, impairing plastoquinone (PQ) pool turnover. Consequently, absorbed light energy is inefficiently converted to chemical energy and lost as heat (high DIo/RC). These findings reveal *E. gracilis* dynamically balances light-energy capture with metabolic resource allocation under varying carbon regimes.

The temporal dynamics of chlorophyll fluorescence parameters (Figure 5 and Figure 6) exhibited fluctuations throughout the cultivation period, which is a common phenomenon in microbial physiology reflecting transient metabolic adaptations to environmental conditions and nutrient availability. However, the values measured at the 10-day endpoint are indeed representative and robust for inter-group comparison for two key reasons. First, by day 10, the cultures had unequivocally entered a stable stationary phase, as evidenced by the plateauing of biomass growth (Figure 1A,B). This physiological stability ensures that the fluorescence parameters reflect a steady-state physiological condition rather than a transient metabolic state. Second, and most importantly, the photosynthetic performance trends observed at day 10 are strongly corroborated by the ultimate yields of key biomolecules measured at the same time point: the high photochemical efficiency in the propanetriol group aligns with its investment in photosynthetic apparatus maintenance under carbon limitation, while the suppressed PSII functionality in the sodium acetate group correlates with its low biomass and pigment yields (Figure 1B, Figure 3 and Figure 4). Therefore, the day 10 time point provides a consistent, physiologically relevant, and practically significant benchmark for comparing the long-term adaptive outcomes induced by the different carbon sources.

Having established the validity of the endpoint comparisons, our data reveal a noteworthy physiological phenomenon: a decoupling between PSII photochemical efficiency and biomass yield under mixotrophic conditions warrants further investigation. Specifically, propanetriol treatment yielded the highest F_v_/F_m_ and F_v_/F_o_ values (Figure 5A,B), indicative of optimal photosynthetic function, yet exhibited the lowest biomass accumulation across all carbon source treatments (Figure 1B). This apparent paradox can be rationalized within the framework of cellular energy allocation and nutrient constraints. The inefficient and slow metabolism of propanetriol likely induced carbon starvation, thereby limiting biosynthetic capacity and growth. In this scenario, the elevated F_v_/F_m_ and F_v_/F_o_ ratios may not reflect high photosynthetic productivity but rather a relative excess of light energy absorption compared to carbon assimilation capacity. As the Calvin cycle is constrained by carbon precursor availability, a greater proportion of absorbed energy is dissipated as fluorescence, leading to increased fluorescence parameters. Consequently, the photosynthetic apparatus is maintained in a state of high photochemical readiness—potentially a stress response to preserve functionality under resource limitation—rather than being utilized for rapid growth. This contrasts with the ethanol or glucose treatments, where efficient carbon metabolism and high growth rates likely resulted in greater non-photochemical quenching of excitation energy, manifesting as slightly lower F_v_/F_m_ values despite significantly higher biomass productivity.

### 3.5. Effects of Different Organic Carbon Sources on the Content of β-1,3-Glucan in E. gracilis

β-1,3-glucan production under organic carbon sources (Figure 3): all treatments with exogenous organic carbon significantly increased the total β-1,3-glucan content in *E. gracilis* cultures compared to the photoautotrophic control (*p* < 0.05). However, the intracellular β-1,3-glucan content (% DW) exhibited a complex relationship with biomass accumulation, showing a general negative correlation as cell density increased [38]. Statistical analysis of paramylon accumulation (Figure 3B) revealed distinct patterns: ethanol and glucose were both highly effective, supporting paramylon content without a significant difference between them. The ethanol group, while achieving the highest biomass accumulation, resulted in the lowest paramylon content among carbon-supplemented groups, indicating preferential carbon channeling into structural biomass rather than storage polymers. This differential partitioning reflects fundamental variations in metabolic strategy: glucose efficiently provides glycolytic ATP and precursors that balance growth with reserve carbohydrate biosynthesis, while ethanol metabolism, operating through coupled TCA cycle/glyoxylate shunt activity, directs carbon flux toward rapid proliferation and chlorophyll synthesis at the expense of storage compound accumulation.

Both glucose and ethanol significantly enhanced both biomass and paramylon accumulation compared to the control, with no statistically significant difference between them in terms of paramylon content (% DW) or final biomass yield. However, glucose showed a stronger tendency toward paramylon storage, while ethanol slightly favored biomass production. Although both carbon sources led to statistically similar endpoint yields, their metabolic routing differed: glucose promoted higher absolute paramylon production per culture volume, while ethanol supported slightly higher chlorophyll b and biomass tendency. This indicates that both carbon sources are highly effective for mixotrophic cultivation of *E. gracilis*, and the choice between them may be influenced by ancillary factors such as pigment yield, cost, or downstream processing requirements. In conclusion, carbon source selection should align with production goals; either glucose or ethanol can be effectively used to boost overall productivity, with the final choice depending on secondary metabolic goals such as pigment co-production or process cost. Metabolic pathway redirection enables precise regulation of growth versus storage product synthesis.

## 4. Conclusions

This study elucidates the distinct regulatory mechanisms by which organic carbon sources govern metabolic partitioning in *E. gracilis*, enabling targeted optimization for industrial applications. Exogenous carbon significantly enhanced biomass and β-1,3-glucan (paramylon) yields compared to photoautotrophic growth. Our results reveal a clear product-oriented specificity: both ethanol and glucose were highly effective drivers for high-density cultivation, supporting comparable and superior biomass accumulation (5.71-fold and 4.42-fold increase, respectively) and paramylon content without a statistically significant difference between them. Ethanol supplementation additionally enhanced chlorophyll b synthesis via synergistic activation of the TCA cycle and glyoxylate shunt. Conversely, glucose notably supported the highest carotenoid content and showed a stronger tendency for paramylon storage. Sodium acetate induced alkaline stress (pH 8.5), suppressing photosynthetic pigments and triggering a protective spherical morphogenesis, while propanetriol enhanced photosystem II efficiency despite limited biomass stimulation. Critically, these carbon sources act as metabolic switches with precise targets: both ethanol and glucose are outstanding for overall biomass and paramylon production, with ethanol directing additional flux toward rapid proliferation and chlorophyll b, while glucose additionally channels carbon into carotenoid and storage carbohydrate synthesis. Our findings establish a mechanistic frame-work for precision carbon source selection—both ethanol and glucose are excellent primary choices; the optimal selection depends on the value of co-products: ethanol for processes benefiting from chlorophyll-rich biomass (e.g., feed) and glucose for applications valuing carotenoid co-production (e.g., nutraceuticals, immunomodulators)—providing a foundational strategy to advance *E. gracilis* as a sustainable cell factory within the circular bioeconomy.

## Figures and Tables

**Table 1:** Effects of different carbon source treatments on the cell aspect ratio.

Treatment Group	Sample Size (Number)	Average Aspect Ratio	Standard Deviation	The Difference with CK (*p* Value)	Median
CK	50	2.513	0.340	——	2.288
Glucose	50	3.697	0.729	*p* < 0.01	3.606
Sodium acetate	50	1.541	0.341	*p* < 0.01	1.168
Ethanol	50	4.224	1.104	*p* < 0.01	4.367
Propanetriol	50	3.593	0.975	*p* < 0.05	3.287

## Data Availability

The original contributions presented in this study are included in the article. Further inquiries can be directed to the corresponding authors.
